# Scaffold attachment factor A (SAF-A) and Ku temporally regulate repair of radiation-induced clustered genome lesions

**DOI:** 10.18632/oncotarget.9914

**Published:** 2016-06-09

**Authors:** Muralidhar L. Hegde, Arijit Dutta, Chunying Yang, Anil K. Mantha, Pavana M. Hegde, Arvind Pandey, Shiladitya Sengupta, Yaping Yu, Patrick Calsou, David Chen, Susan P. Lees-Miller, Sankar Mitra

**Affiliations:** ^1^ Department of Radiation Oncology, Houston Methodist Research Institute, Houston, TX, USA; ^2^ Houston Methodist Neurological Institute, Houston, TX, USA; ^3^ Weill Medical College of Cornell University, Ithaca, NY, USA; ^4^ Department of Biochemistry and Molecular Biology, University of Texas Medical Branch, Galveston, TX, USA; ^5^ Department of Biochemistry & Molecular Biology, University of Calgary, Calgary, Canada; ^6^ Institut de Pharmacologie et de Biologie Structurale, CNRS, Université de Toulouse-Université Paul Sabatier, Equipe Labellisée Ligue contre le Cancer, Toulouse, France; ^7^ UT Southwestern Medical Center, Dallas, TX, USA; ^8^ Center for Animal Sciences, School of Basic and Applied Sciences, Central University of Punjab, Bathinda, Punjab, India

**Keywords:** SAF-A/hnRNP-U, Ku, DNA-PK, BER, DSB repair

## Abstract

Ionizing radiation (IR) induces highly cytotoxic double-strand breaks (DSBs) and also clustered oxidized bases in mammalian genomes. Base excision repair (BER) of bi-stranded oxidized bases could generate additional DSBs as repair intermediates in the vicinity of direct DSBs, leading to loss of DNA fragments. This could be avoided if DSB repair via DNA-PK-mediated nonhomologous end joining (NHEJ) precedes BER initiated by NEIL1 and other DNA glycosylases (DGs). Here we show that DNA-PK subunit Ku inhibits DGs via direct interaction. The scaffold attachment factor (SAF)-A, (also called hnRNP-U), phosphorylated at Ser59 by DNA-PK early after IR treatment, is linked to transient release of chromatin-bound NEIL1, thus preventing BER. SAF-A is subsequently dephosphorylated. Ku inhibition of DGs *in vitro* is relieved by unphosphorylated SAF-A, but not by the phosphomimetic Asp59 mutant. We thus propose that SAF-A, in concert with Ku, temporally regulates base damage repair in irradiated cell genome.

## INTRODUCTION

Ionizing radiation (IR) as well as radiomimetic drugs induce clusters of damage in the genome, including most cytotoxic double-strand breaks (DSBs), as well as single-strand breaks (SSBs) with nonligatable termini, bi-stranded clusters of abasic (AP) sites, and oxidized bases [1, 2]. To restore genomic integrity, DSBs activate signalling cascades that begin with the binding of the Mre11/Rad50/Nbs1 (MRN) complex to the DSBs followed by activation of the protein kinase ATM, which subsequently activates the cell cycle checkpoint and DNA repair pathways [3–5]. DSB repair occurs *via* homologous recombination (HR) in S/G_2_ cells and *via* nonhomologous end joining (NHEJ) in all cells [4, 6, 7]. NHEJ, the predominant repair process in all cells, is initiated by the binding of Ku (Ku70/Ku80 heterodimer) subunit of DNA-dependent protein kinase (DNA-PK) to the DSB site, which then recruits the catalytic subunit (DNA-PKcs). DNA-PK in addition to phosphorylating many downstream targets enables formation of a large NHEJ complex comprising DNA ligase4/XRCC4/XLF that joins the DSBs after their end processing [6]. The base lesions and AP sites are repaired *via* the base excision/SSB repair (BER/SSBR) pathway and involves formation of SSB intermediates, which like the IR-induced direct SSBs bind PARP-1. PARP-1 in turn recruits DNA glycosylases (DGs), AP-endonuclease 1 (APE1), DNA polymerases, and DNA ligase 3α (Lig3α)/XRCC1 [8, 9]. While NHEJ is the major pathway for DSB repair [10], alternative NHEJ (Alt-EJ) involving PARP-1 and the BER machinery may also repair DSBs, including those generated during the repair of bi-stranded oxidized lesion clusters; however its contribution to DSB repair is not very clear [11].

How multiple repair pathways, particularly NHEJ and BER, are coordinated in repairing IR-induced damage clusters in the mammalian genome have not been investigated. The SSB intermediates generated during repair of oxidized bases and AP sites, in the proximity of an unrepaired DSB, could cause additional DNA sequence loss [12, 13]. Furthermore, bi-stranded base lesions/AP sites and SSBs could produce secondary DSBs [14–18]. Hence, we hypothesized that NHEJ precedes BER.

Recent studies have documented involvement of non-canonical proteins, in particular, the family of RNA/DNA binding proteins, e.g., hnRNPs in the repair of both oxidized lesions and DSBs [19, 20]. While hnRNP-U is primarily known for its role in mRNA processing and transport [21], it was independently identified and named scaffold attachment factor (SAF)-A, on the basis of its strong binding to nuclear scaffold/ matrix with affinity for A-T-rich DNA [22]. This 90 kDa protein is the largest member of the abundant hnRNP family. Interestingly, hnRNP-U/SAF-A was also found to be associated with WT1 (Wilms tumor 1) protein and suggested to be a potential Wilms tumor gene [23]. It interacts with MDM2, an E3 ubiquitin ligase involved in degradation of p53, a central player in DNA damage response. We previously characterized the presence of SAF-A/hnRNP-U in the immunoprecipitates (IPs) of both NEIL1 and NEIL2 and its functional implication in BER [19, 20]. More recent studies have documented that irradiation induces its phosphorylation at Ser59 by DNA-PK, followed by its dephosphorylation, presumably after completion of DSB repair [24, 25]. However, the possible involvement of hnRNP-U/SAF-A in radiation-induced clustered damage repair has not been explored. Polo-like kinase 1 also phosphorylates SAF-A at Ser59 which has been implicated in accurate mitosis [26].

In this report, we demonstrate that Ku negatively regulates BER by inhibiting base excision/strand-scission activity of all oxidized base-specific DGs at IR-induced clustered damage sites to allow the completion of DSB repair *via* NHEJ. SAF-A then reverses inhibition to activate BER, which is regulated by its IR-responsive phosphorylation-dephosphorylation events. Our experimental observations are consistent with the temporal regulation of NHEJ and BER at IR-induced damage clusters, which is critical for maintaining genomic fidelity in human cells.

## RESULTS

### BER contributes to radioresistance in human cells

**Figure 1:**
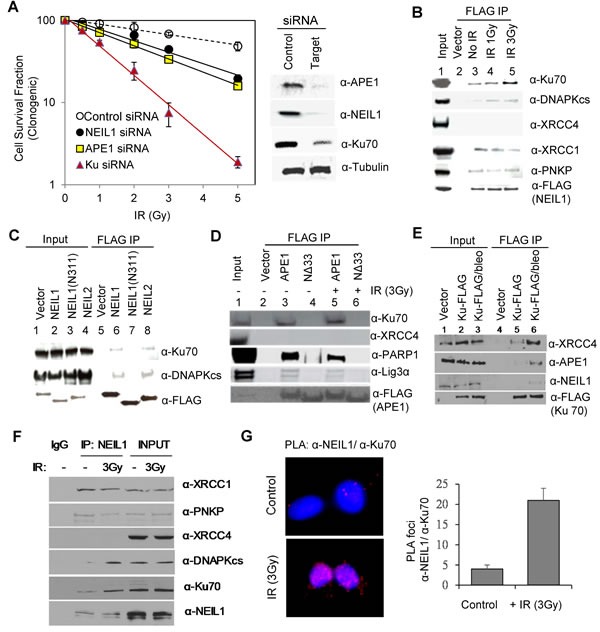
Crosstalk between BER and DSB repair after irradiation **A.** Loss of APE1 or NEIL1 causes moderate radiosensitization. For clonogenic survival analysis (left panel), HEK293 cells were irradiated at 48 h after transfection with siRNAs. Immunoblotting (right panel) shows depletion of target proteins. **B.** The FLAG-NEIL1 co-IP in HEK293 cells revealed radiation-associated increase in association of Ku, DNA-PKcs, and PNKP but not of XRCC4 with NEIL1. **C.** The IP of WT APE1 but not of the NΔ33 mutant (lacking APE1's common interaction domain) contained Ku and BER/SSBR proteins, The FLAG IPs were isolated from HEK293 cells after transfection with FLAG-tagged WT APE1 or the NΔ33 mutant. **D.** The FLAG-NEIL1/2 IP contained DNA-PKcs and Ku, but the IP of NEIL1(N311) mutant that lacks the common interaction domain (aa312-389) did not contain DNA-PKcs or Ku, underscoring the specificity of Ku interaction. **E.** The FLAG-Ku IP contained XRCC4, APE1, and NEIL1, whose levels increased after bleomycin treatment. **F.** Endogenous NEIL1 co-IP contains Ku70, DNA-PKcs after irradiation, unlike PNKP and XRCC1, which are constitutively associated. **G.** PLA analysis confirms in-cell association of NEIL1 with Ku, which was enhanced after IR treatment (> 25 cells were counted for the bar graph).

To test the role of BER in the repair of IR-induced clustered genome damage, we analysed radiosensitivity of BER-deficient human cells using clonogenic cell survival assay. siRNA-mediated depletion of NEIL1 or APE1 caused a modest reduction in survival of irradiated HEK293 cells (Figure 1A) as compared to that of IR-treated NHEJ-deficient cells [4, 27, 28]. Moderate radioprotective role of BER enzymes was consistently observed in mouse fibroblasts [29, 30]. Furthermore, varying degrees of radiosensitivity associated with BER and NHEJ protein deficiencies [31] suggest generation of distinct subgroups of IR-induced lesions that independently and additively affect cell survival.

### Early NHEJ proteins interact with BER enzymes in irradiated cells

We next tested for the presence of crosstalk between BER and DSB repair machinery in cells after irradiation. Co-immunoprecipitation (co-IP) analysis with FLAG antibody (Ab) from nuclear extract of HEK293 cells stably expressing FLAG-NEIL1, after degradation of nucleic acids, revealed stable binding of early NHEJ proteins Ku70 and DNA-PKcs to NEIL1, whose levels in the IP significantly increased after irradiation (Figure 1B). The absence of Ku in the IP of NEIL1 C-terminal domain (CTD) deletion mutant (N311)-FLAG indicated that Ku interaction requires the CTD [which was previously implicated for most of NEIL1's DNA-independent, binary interaction [32]] (Figure 1C). The presence of Ku in the NEIL1 IP from ethidium bromide (EtBr)-treated cell extracts further confirmed lack of involvement of nucleic acids in the NEIL1-Ku interaction (data not shown). The IP of NEIL2, a related DG that serves as the primary back up for NEIL1 [33], also contained Ku70 and DNA-PKcs (Figure 1C). Similarly, the FLAG-(WT)-APE1 IP contained Ku70, along with the other BER proteins PARP1 and Lig3α; none of these was detected in the IP of N-terminal deletion mutant APE1 (NΔ33) (Figure 1D). Analogous to the CTD of NEIL1, the N-terminal domain of APE1 provides the common interaction interface for the BER proteins [34]. XRCC4, a key NHEJ protein, was not detected in the IP of either NEIL1 or APE1. These interactions were also enhanced after treatment with radiomimetic bleomycin. The FLAG IP from HEK293 cells transiently expressing FLAG-Ku provided confirmatory evidence for Ku's interaction with the BER proteins NEIL1 and APE1, as well as the NHEJ protein XRCC4 (Figure 1E). While lack of association of FLAG-NEIL1(N311) polypeptide with Ku revealed the specificity of interaction, in order to exclude any possible artefacts of ectopic expressions, we further confirmed their *in cell* association and its enhancement after irradiation by analysing endogenous co-IP with NEIL1 Ab (Figure 1F). Finally, we confirmed our conclusion by using proximity ligation assay (PLA; Figure 1G).

### Ku inhibits DNA glycosylases and APE1 *via* direct interaction

**Figure 2:**
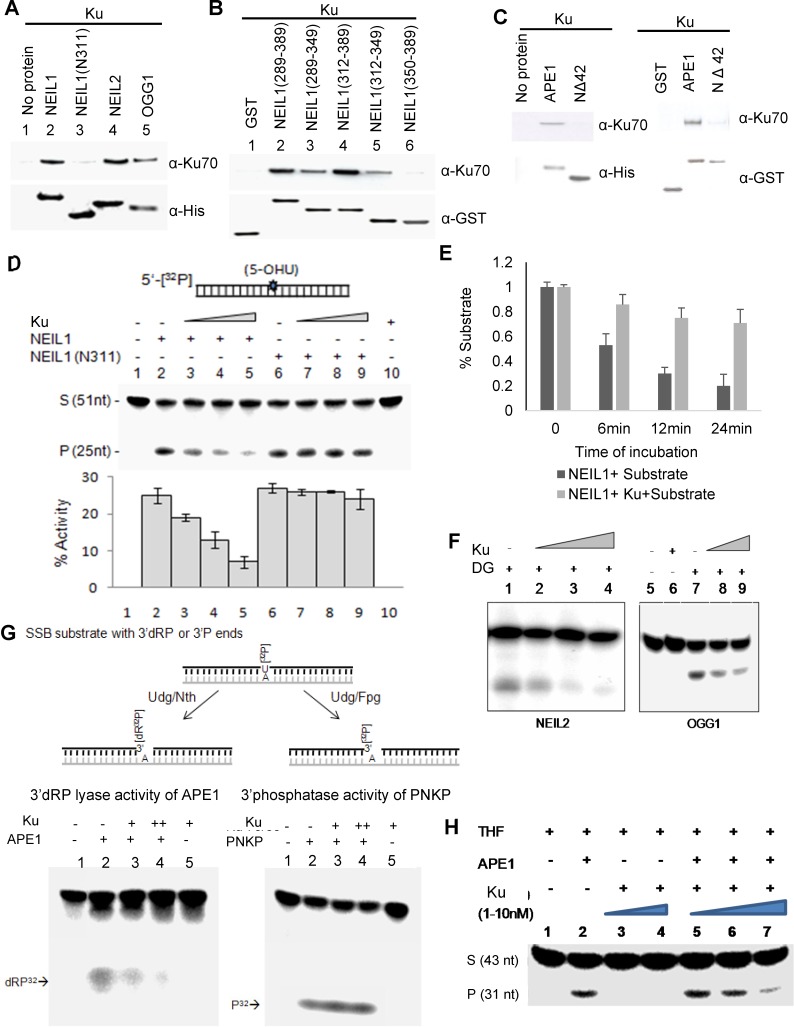
Ku inhibits DGs direct interaction **A.** Direct interaction of Ku with NEIL1 (but not the N311 mutant), NEIL2, and OGG1 was confirmed by His-affinity pull down analysis using purified proteins in the absence of DNA. **B.** GST pull down analysis showing that NEIL1 aa289-349, but not aa350-389, was needed for Ku binding. **C.** The binary interaction of Ku with APE1 (but not the NΔ42 mutant) is shown by His-affinity pull down analysis. **D.** Dose-dependent inhibition of WT NEIL1 (1nM, lane 2) by Ku (0.1, 0.5 and 1nM; lanes 3-5), but not by the N311 mutant (1nM, lane 6-9) is shown with a 5-OHU-containing 51-mer duplex substrate (25nM). **E.** Time kinetics of Ku inhibition of NEIL1 activity. **F.** NEIL2 and OGG1 are also inhibited by Ku at similar experimental conditions. **G.** A 26-nt oligo containing U at the 5′ terminus was labelled with γ^32^P-ATP using T4-PNK and annealed with a 25-nt proximal sequence and a 51-nt complementary oligo. The duplex was digested with Udg/Nth or Udg/Fpg to generate a strand break with a 3′dRP or 3′P end, respectively (top). Ku inhibits 3′dRPase activity of APE1 (bottom left panel), but not the 3′phosphatase activity of PNKP (bottom right panel). **H.** Ku inhibition of AP endonuclease activity of APE1.

To assess the impact of Ku's interaction with BER-initiating enzymes, we examined their *in vitro* interaction using purified, recombinant proteins. Affinity co-elution of Ku with the His-tagged DGs NEIL1, NEIL2, and OGG1, which were pre-bound to Ni-NTA magnetic beads, confirmed Ku's direct binding to the DGs. The absence of Ku binding to the NEIL1 (N311)-His tag deletion protein was predicted and supports the *in-cell* data (Figure 2A). With pulldown assay using GSH column bound, GST-tagged NEIL1 CTD peptides aa289-389, aa289-349, aa312-389, aa312-349, or aa350-389, we mapped Ku's interacting region within the CTD of NEIL1. The absence of aa350-389 did not affect binding, which suggests that Ku binding requires aa289-349 residues of NEIL1 (Figure 2B). Similarly, binary interaction between Ku and APE1 was confirmed using His and GST pull down assays using His/GST-tagged WT APE1 or the NΔ42 mutant (Figure 2C).

We then tested the impact of Ku's interaction on the activity of DGs or APE1 using 5-OHU or THF-containing duplex oligonucleotide substrates, respectively, as previously described [35]. The Ku heterodimer inhibited base excision/strand cleavage activity of full-length NEIL1 but not of the N311 mutant in a dose-dependent manner (Figure 2D). This result indicates that inhibition of NEIL1 by Ku requires its physical interaction with NEIL1. Furthermore, while the dose-dependent NEIL1 inhibition by Ku was quantitated after 20 min incubation, we confirmed linearity of Ku's inhibition of NEIL1 by measuring activity at 0, 6, 12 and 24 min (Figure 2E). Similarly, Ku inhibits other oxidized base-specific DGs NEIL2 and OGG1 as well (Figure 2F). Furthermore, Ku inhibits the 3′dRP lyase activity of APE1 at an SSB site in a duplex oligonucleotide, as well as AP endonuclease activity with a THF-containing substrate (Figure 2G, 2H). In contrast, Ku did not affect the 3′ phosphatase activity of polynucleotide kinase-phosphatase (PNKP) at an SSB site (Figure 2G). It should be noted that PNKP is required both in NEIL-initiated BER and NHEJ [36, 37].

### Distinct association of SAF-A with NHEJ and BER complexes is mediated by SAF-A's DNA-PK-dependent phosphorylation

**Figure 3:**
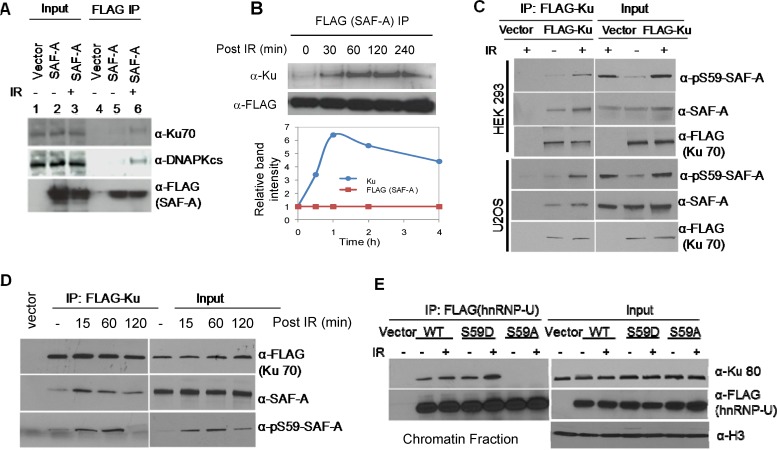
*In cell* association of Ku with SAF-A is regulated by SAF-A phosphorylation at Ser59 **A.** Presence of Ku and DNA-PK increased post irradiation (3Gy, 1 h) in the SAF-A IP isolated from HEK293 cells. **B.** Dynamics of Ku association with SAF-A after irradiation; Ku level was measured in the FLAG-SAF-A IP from HEK293 cells. **C.** Increased association of SAF-A and pS59-SAF-A in the FLAG-Ku IP after irradiation of HEK293 (upper panel), and U2OS cells (bottom panel). **D.** Kinetics of association of SAF-A and pS59-SAF-A with FLAG-Ku in HEK293 cells. **E.** Ku binds to WT SAF-A and the phosphomimetic S59D mutant in chromatin but not to the non-phosphorylatable S59A mutant.

SAF-A was previously implicated in the IR-induced genome damage response [24, 25] and was shown to be phosphorylated at Ser59 by DNA-PK in irradiated cells. While SAF-A's role in DSB repair was not investigated, we showed earlier that it stimulates NEIL1-initiated BER, and thereby enhances repair of ROS-induced oxidative base damage [20, 38]. In the present study, we observed association of SAF-A with both NHEJ and BER proteins. Both Ku70 and DNA-PKcs were detected in the SAF-A-FLAG IP and their levels were significantly increased after irradiation (Figure 3A). Furthermore, the Ku-SAF-A *in-cell* complex reached the peak level at 1 h post-irradiation (Figure 3B). Next, we examined the phosphorylation status of Ku-bound SAF-A. The FLAG IP from both HEK293 and U2OS cells transiently expressing Ku-FLAG contained p-Ser59-SAF-A, whose level increased after irradiation (Figure 3C). Kinetic analysis showed the presence of p-Ser59-SAF-A in the Ku-FLAG IP as early as 15 min post-irradiation, which peaked at 1 h before returning to the basal level at 4 h (Figure 3D). These results strongly suggest that phosphorylation of SAF-A regulates its interaction with Ku. When FLAG-tagged phosphomimetic S59D and non-phosphorylable S59A mutants of SAF-A were transiently expressed in HEK293 cells, the presence of Ku was observed in the IP of both WT SAF-A and the S59D mutant, but not in that of the S59A mutant. These results further support the conclusion that SAF-A, only when phosphorylated, stably associates with Ku (Figure 3E). Collectively, these data are consistent with the scenario that SAF-A, phosphorylated by DNA-PK in response to IR-induced DNA damage, remains bound to the NHEJ complex until completion of DSB repair, and is subsequently dephosphorylated.

### IR induces SAF-A phosphorylation and blocks BER initiation by transient dissociation of NEIL1 from chromatin

**Figure 4:**
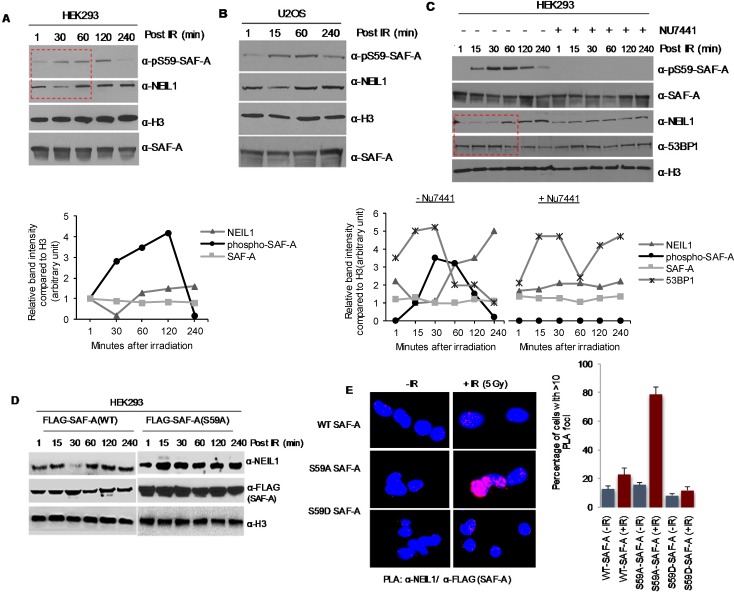
SAF-A phosphorylation by DNA-PK is linked to transient dissociation of NEIL1 from chromatin **A**-**B.** Enhanced pS59 SAF-A formation in the HEK293/ U2OS cell chromatin after irradiation. Chromatin-bound NEIL1 was released at ~15-30 min after IR exposure and restored after 1h, shown in quantitative histogram (*lower panel, for HEK293*). **C.** Chromatin-bound NEIL1 level was not altered by pretreatment with the DNA-PKcs inhibitor NU7441, which prevented SAF-A phosphorylation. NU7441 (10 μM) pretreatment for 2 h followed by exposure to 3 Gy X-rays and chromatin extraction from cells harvested at the indicated times. NEIL1's transient dissociation from chromatin coincided with increase in the 53BP1 level, consistent with NHEJ repair of overt DSBs, while BER was delayed. Quantitative histograms shown in *lower panels*. **D.** WT SAF-A, but not the non-phosphorylatable S59A mutant, negatively regulated chromatin-bound NEIL1. HEK293 cells were transfected with 3′ UTR hnRNP-U siRNA and FLAG-hnRNP-U WT or S59A, followed by irradiation. **E.** PLA analysis revealed increased interaction of SAF-A(S59A), but not the WT or S59D mutant with NEIL1 after irradiation. HEK293 cells were transfected with FLAG-tagged WT, S59A, or S59D SAF-A plasmid, and 48 h later, were irradiated (3 Gy). The PLA was performed for NEIL1 and FLAG -SAF-A after further incubation for 30 min.

Because SAF-A interacts with both NHEJ and BER proteins [19, 20], we explored its possible involvement in the pathways for repair of clustered damage in chromatin. NHEJ of DSBs directly induced by IR occurs in the 15-60 min window after irradiation, as indicated by an increase in 53BP1 level in the chromatin (Figure 4C), consistent with the previous studies [4]. NEIL1's transient dissociation from chromatin was observed in both U2OS and HEK293 cells at 15-30 min post irradiation, at about the same time when SAF-A was phosphorylated (Figure 4A-4C). NEIL1 is restored to chromatin at 1 h after irradiation when DSB repair is mostly completed, as further revealed by 53BP1 level. This suggests a causal link between SAF-A phosphorylation and NEIL1's dissociation from chromatin, in order to prevent BER initiation and allow DSB repair by NHEJ. To further support this possibility, HEK293 cells were treated with the DNA-PK inhibitor NU7441, and the chromatin fraction was analysed at various times after irradiation (Figure 4C). Dissociation of NEIL1 from chromatin was prevented in DNA-PK inhibited cells at 30 min post irradiation. Furthermore, in HEK293 cells with 3′UTR-specific siRNA mediated knockdown of endogenous SAF-A together with ectopic expression of WT SAF-A or the non-phosphorylable mutant, release of chromatin-bound NEIL1 was observed only in WT cells (Figure 4D). It is important to point out that while NEIL1's dissociation from chromatin fraction tightly correlated with SAF-A phosphorylation, peaking at 30 min after irradiation, NEIL1 re-association with chromatin occurred at 1h post IR but SAF-A dephosphorylation followed a slower kinetics, completing after 2-4h. This suggests the presence of additional mechanisms regulating NEIL1's re-association with chromatin. Our recent data show that NEIL1 is acetylated by p300, which is required for its chromatin binding. Here we show that kinetics of NEIL1's dissociation from chromatin post-irradiation correlates with its deacetylation (Supplemental Figure S1). Thus it is likely that NEIL1 acetylation along with other factors are also involved in this regulation.

**Figure 5:**
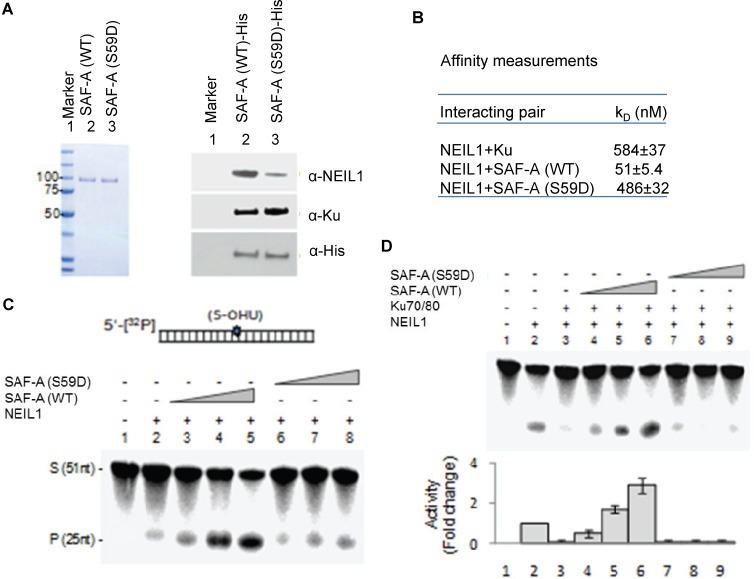
WT SAF-A but not the S59D mutant overrides NEIL1 inhibition by Ku **A.** Purification of WT and S59D SAF-A to near homogeneity (left panel after Coomassie staining). *In vitro* His-pull down of untagged NEIL1 or Ku by His-tagged SAF-A (WT *vs*. S59D) bound to Ni-beads. NEIL1 binding to the S59D mutant was weaker than binding to WT SAF-A (right panel). **B.** Affinity measurement of NEIL1 for Ku, WT SAF-A and the S59D mutant by fluorescence analysis where 10-fold lower affinity was observed for the phosphomimetic mutant than for WT SAF-A. **C.** WT SAF-A (0.1, 05, 1nM; lanes 3-5), but not the S59D mutant (0.1, 05, 1nM; lanes 6-8), stimulated NEIL1 (1nM; lane 2) activity, and **D.** reversed Ku-dependent inhibition.

Additionally, PLA analysis showed only modest increase in NEIL1's *in-cell* interaction with SAF-A in HEK293 cells at 30 min after irradiation. In contrast, significantly higher number of PLA foci were observed in HEK293 cells expressing FLAG-SAF-A(S59A), suggesting its elevated association with NEIL1 (Figure 4E). These data are consistent with non-phosphorylated SAF-A's higher affinity for NEIL1 (Figure 5B) and lower affinity for Ku (Figure 3E). These results thus provide strong support for temporal regulation of NHEJ and BER after treatment with IR, coordinated by SAF-A phosphorylation and regulating release of chromatin-bound NEIL1 (Figure 4E).

### WT SAF-A relieves Ku inhibition of NEIL1 *in vitro*

To gain further mechanistic insight about the impact of SAF-A phosphorylation on BER, NEIL1's affinity for WT SAF-A *versus* the S59D mutant was measured by fluorescence and affinity co-elution analyses. *In vitro* His-affinity co-elution of recombinant NEIL1 with His-SAF-A was observed, while co-elution with the S59D mutant was observed at a significantly lower level (Figure 5A). In contrast, Ku binding to the S59D mutant was higher compared to the WT SAF-A polypeptide. Fluorescence analysis confirmed this, in which NEIL1's affinity for the WT protein (non-phosphorylated) was found to be about 10-fold higher than for the phosphomimic mutant (Figure 5B). Together, these results support the scenario that phosphorylated SAF-A, as a component of the NHEJ complex, ensures Ku inhibition of NEIL1 and may also contribute to NEIL1's dissociation from chromatin in order to prevent BER. SAF-A, dephosphorylated after completion of NHEJ, stimulates NEIL1 even in the presence of Ku, thus acting as a molecular switch for the NHEJ-to-BER transition.

We then investigated the effect of SAF-A phosphorylation on NEIL1's DG activity *in vitro*. WT SAF-A stimulated NEIL1 activity, confirming earlier studies [20] (Figure 5C), and unlike the S59D mutant overrode Ku inhibition of NEIL1 (Figure 5D). As Ku associates with both SAF-A and NEIL1, we next tested whether Ku forms distinct complex(es) with SAF-A and NEIL1 for its NHEJ and BER role by fractionation of U2OS cell nuclear extracts before and after irradiation, on a Sephacryl-S300 gel filtration column (Supplemental Figure S2). Consistent with our co-IP analysis, Western analysis shows that Ku elutes in multiple distinct complexes with SAF-A (> 1000kDa) or NEIL1 (~600kDa).

### SAF-A plays a role in IR-induced damage repair and its radioprotective function requires Ser59 phosphorylation

**Figure 6:**
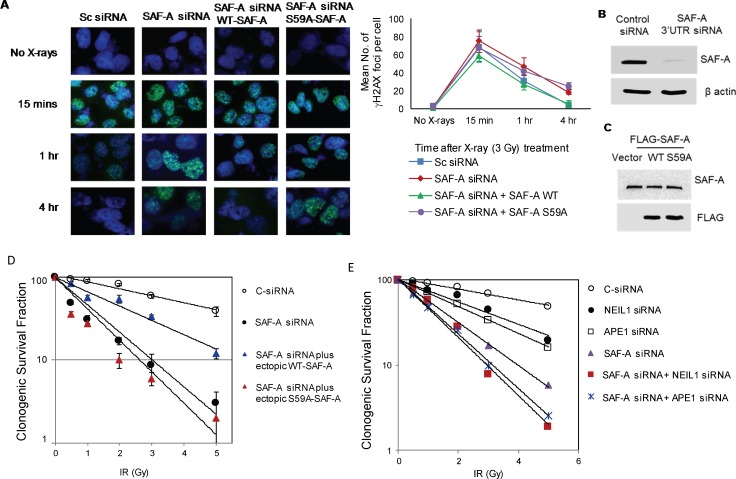
SAF-A's radioprotective function requires its S59 phosphorylation and its additive effect with APE1 and NEIL1 **A.** HEK293 cells were transfected with 3′UTR-specific SAF-A siRNA alone or in combination with plasmids for WT or S59A FLAG-SAF-A. After irradiated at 48 h (3Gy), these cells were analysed microscopically for γH2AX foci kinetics or clonogenic survival analysis. **B.** Immunoblotting analysis shows downregulation of SAF-A with 3′UTR siRNA in U2OS cells. **C.** Transient expression of FLAG-SAF-A(WT), FLAG-SAF-A(S59A), FLAG-SAF-A(WT/S59A) in U2OS cells. **D.** Clonogenic survival of cells transfected with control siRNA, SAF-A 3′UTR siRNA, or SAF-A 3′UTR siRNA plus FLAG-WT/S59A SAF-A plasmids. Cells were irradiated with different dose of x-rays as indicated and three hundred cells were plated in triplicate on 6-cm dishes. After 7-10 days, individual colonies were fixed, stained, and counted for the survival assay. **E.** SAF-A siRNA-mediated singly or doubly deficient (in combination with NEIL1 or APE1 siRNA) HEK293 cells, along with the control, were irradiated at 48 h after transfection, and 300 cells were plated in triplicate for the survival assay as before.

We previously showed that SAF-A activates NEIL1-initiated BER particularly after oxidative stress [20]. Here, we investigated the impact of SAF-A depletion on the repair of IR-induced DSB damage by γ-H2AX foci disappearance and comet analysis. Significant delay in the disappearance of γ-H2AX foci from irradiated (3Gy) U2OS cells was observed after SAF-A depletion compared to that in cells expressing control (scrambled) siRNA (Figure 6A, 6B, 6C). Furthermore, the slower DSB repair kinetics was rescued by ectopic expression of WT SAF-A but not the nonphosphorylatable S59A-SAF mutant (Figure 6A, 6B, 6C). Consistent with this observation, comet analysis showed comparable level of strand breaks at 4h post irradiation in SAF-A depleted cells under alkaline *vs*. neutral condition (Supplemental Figure S3). Furthermore, in view of BER proteins’ modest contribution to radioresistance (Figure 1A), we examined the role of SAF-A in cell survival after irradiation. 3′UTR siRNA-mediated depletion of SAF-A caused moderate decrease in clonogenic survival, which was partially rescued by ectopic WT SAF-A, but not by the S59A mutant (Figure 6D). Combined depletion of BER enzymes and SAF-A was then checked for additive sensitivity. Depletion of both NEIL1 and APE1 together with SAF-A caused higher radiosensitivity than depletion of SAF-A alone (Figure 6E). These results are consistent with SAF-A's critical role in protection from radiation-induced genome damage.

## DISCUSSION

IR-induced genotoxicity results from the formation of clustered damage in the genome that include overt DSBs together with oxidized base lesions or AP sites, and SSBs. Elucidating the repair processes for IR or radiomimetic drug-induced genome damage is important for improved cancer therapy and protection from accidental or therapeutic radiation. In mammalian cells, the major DSB repair pathways HR and NHEJ are biochemically distinct with diverse substrate requirements, and these follow different kinetics in a cell cycle-dependent manner [39]. NHEJ is the predominant repair pathway for DSBs induced by irradiation or radiomimetic drugs. NHEJ-deficient mammalian cells are extremely sensitive to X- and γ-rays and accumulate unrepaired DSBs as a function of radiation dose [40].

While numerous studies have focused on the repair of IR-induced DSBs by the NHEJ pathway, bi-stranded, non-DSB lesion clusters, which include oxidized bases, SSBs and AP sites that are generated at a much higher level than DSBs, have received little attention. It is likely that the deleterious effects of radiation are primarily caused by clustered damage rather than overt DSBs alone [1]. Earlier studies suggested that overt DSBs in irradiated cells are re-joined first, followed by repair of clustered non-DSB damage at a slower rate [41]. However, how such distinct repair processes are coordinated has not been investigated. As demonstrated in this study and elsewhere [15], non-DSB damage, primarily repaired *via* the BER/SSBR pathway, also contributes to the radiosensitivity of tumor cells. Because BER generates intermediate SSBs, repair of IR-induced bi-stranded damage clusters could create additional DSBs and lead to loss of genomic sequences. Furthermore, these additional strand breaks in the vicinity of an overt DSB could cause large deletions [17, 42].

Earlier studies suggested that NHEJ alone is not sufficient to handle radiation-induced damage clusters in mammalian cells [41]. Okayasu and his colleagues [43] showed by measuring chromosome fragmentation and γH2AX foci formation that NHEJ inadequately repairs clustered damage. Recent studies showed that high-energy IR kills more cells than low-energy IR at the same dose level because of inefficient Ku-dependent NHEJ repair, which was subsequently confirmed in NHEJ-deficient mice [44].

**Figure 7:**
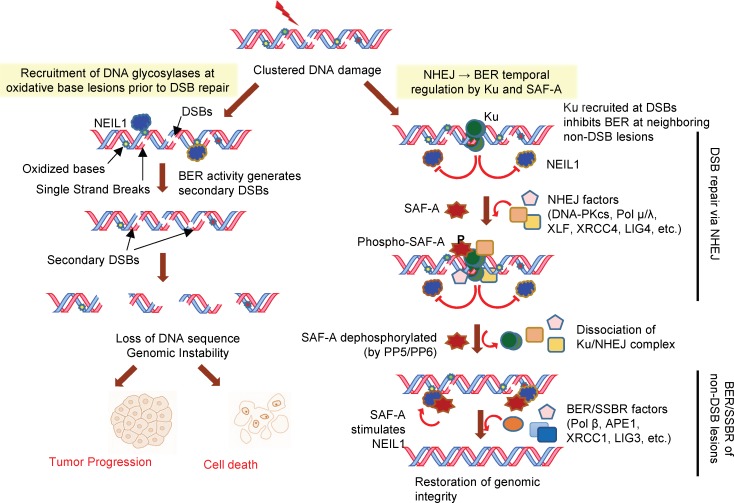
Model of temporal regulation of IR-induced clustered damage in human genome BER activity at oxidative base lesions at clustered DNA damage could lead to generation of secondary DSBs. Such secondary DSBs generated prior to repair of existing DSBs could lead to loss of genomic sequence. Repair of DSBs in the IR-induced damage cluster is initiated by Ku recruited at DSB, which then assembles the NHEJ complex after recruiting DNA-PKcs. Recruitment of Ku at DSBs also inhibits BER of oxidized bases and AP sites (*this study*). Early phosphorylation of SAF-A at Ser59 by DNA-PK correlates with transient dissociation of NEIL1 from chromatin to prevent BER initiation. Residual DGs in chromatin are inhibited by Ku while NHEJ occurs. After completion of DSBR, SAF-A is dephosphorylated, relieving Ku inhibition of BER, and restoring NEIL1 levels in chromatin. Ku together with SAF-A thus act as a molecular switch for NHEJ→BER transition.

The mechanisms of crosstalk between BER and NHEJ at the damage clusters, to prevent large loss of DNA sequences, is not known. We show in this study that coordinated NHEJ repair of overt DSBs precedes repair of neighboring oxidized bases in irradiated cells. The sequential NHEJ→BER model (Figure 7) is supported by three key observations: (a) Ku immunocomplexes in human cells contain BER proteins including DGs and APE1, all of which are directly inhibited by Ku via binary interaction. This observation is consistent with prior reports of Alt-NHEJ suppression by DNA-PK/Ku [11, 45, 46]. (b) BER inhibition by Ku is alleviated by SAF-A during NHEJ, but not by the phosphorylated protein [24, 25]. It is thus likely that after NHEJ completion, SAF-A acts as a molecular switch for the NHEJ→BER transition. (c) Consistent with this model, SAF-A via its S59 phosphorylation, regulates transient dissociation of chromatin-bound NEIL1 soon after irradiation, presumably to prevent BER initiation and also allow overt DSB joining *via* NHEJ. Prevention of dissociation of chromatin-bound NEIL1 by DNA PK inhibition, SAF-A depletion or ectopic non-phosphorylatable S59A mutant, supports this scenario. Furthermore, release of chromatin-bound NEIL1 correlates well with the kinetics of SAF-A phosphorylation and NHEJ of overt DSBs after IR exposure [4, 47], suggesting tight regulation. However, while the NEIL1 level in chromatin is restored 1 h after IR, SAF-A dephosphorylation requires 2-4 h, suggesting the recovery may involve additional factors or mechanisms.

Once overt DSBs are repaired *via* NHEJ, repair of non-DSB lesions *via* BER/SSBR may be initiated, which involves PARP-1 and XRCC1 [4]. Additional DSBs generated during the repair of bi-stranded damage clusters [14–18] are likely repaired exclusively by Alt-EJ because the presence of PARP-1 inhibits Ku recruitment and thus NHEJ [4, 48]. Consequently, the hierarchy of NHEJ→ BER/Alt-EJ prevents greater loss of genomic sequences that would otherwise occur with concurrently active BER and NHEJ.

Our studies thus not only underscore the contribution of BER to radioresistance of human cells, but also establish the critical importance of temporal regulation of NHEJ and BER. Because of the extensive use of IR and radiomimetic drugs in cancer therapy, these results may help to identify novel BER/Alt-NHEJ targets along with NHEJ targets for simultaneous radiosensitization of tumors.

## MATERIALS AND METHODS

### Cell culture

The human embryonic kidney HEK293 (ATCC # CRL-1573) and osteosarcoma U2OS (ATCC # HTB-96) cell lines were grown in Dulbecco's Modified Eagle's medium (DMEM; Gibco, Carlsbad, CA) supplemented with 10% fetal bovine serum (FBS) and 100μgml penicillinstreptomycin (Thermo Fisher, Waltham, MA) at 37°C in the presence of 5% CO_2_ and 95% relative humidity. Zeocin (100 μg/ml) was supplemented in the medium for culturing HEK293 cells stably expressing FLAG-NEIL1.

### Subcellular fractionation

The chromatin fraction was prepared as previously described [49]. Briefly, after harvesting by scraping culture dishes, the cells were lysed in a cytoplasmic buffer [10 mM Tris-HCl pH 7.9, 0.34 M sucrose, 3 mM CaCl_2_, 2 mM Mg(OAc)_2_, 0.1 mM EDTA, 1mM DTT, 0.1% Nonidet P-40, and protease inhibitor mixture (Roche Applied Science)], and then centrifuged at 3500 × g for 15 min at 4°C. The pellet was suspended in nuclear lysis buffer (20 mM HEPES pH 7.9, 1.5 mM MgCl_2_, 3 mM EDTA, 1 mM DTT, 10% glycerol, 0.5% Nonidet P-40, and the protease inhibitor mixture), and centrifuged at 14000 rpm for 10 min to separate the soluble nuclear fraction from the chromatin pellet. The pellet was dispersed in chromatin lysis buffer (150 mM HEPES pH 7.9, 1.5 mM MgCl_2_, 150 mM potassium acetate, 10% glycerol, and protease inhibitor mixture), digested with 0.15 unit/μl of benzonase or EtBR at 37°C for 30 min to degrade or remove nucleic acids, and centrifuged at 14000 rpm for 15 min at 4°C. The supernatant chromatin fraction was used for analysis.

### Co-immunoprecipitation (Co-IP) assay

For co-IP assay, the lysates of chromatin or the nuclear lysates were immunoprecipitated for 3 h at 4°C with FLAG M2 Ab-bound agarose beads (Sigma, St. Louis, MO). The beads were washed three times with 0.5 ml cold Tris-buffered saline (TBS), eluted in 40 μl Laemmli buffer. The eluate was separated by SDS-PAGE for immunoblotting with appropriate Abs.

### Antibodies

Anti-hnRNP-U/SAF-A, anti-APE1, anti-Ku 70, anti-DNA-PKcs, anti-beta tubulin, anti-beta actin, and anti-Lig3 Abs were purchased from Abcam (Cambridge, MA). Anti-APE1 was obtained from Santa Cruz Biotechnology (Santa Cruz, CA). Anti-PARP1, anti-Ku 80, anti-XRCC1, anti-XRCC4, anti-GST, anti-His, anti-H3, and anti-NBS1 were purchased from Cell Signaling (Beverly, MA). Anti-FLAG Ab was purchased from Sigma (St. Louis, MO). The rabbit anti-NEIL1 and anti-pS59 SAF-A Abs were custom-generated [26, 33, 50].

### Purification of recombinant proteins

Recombinant wild type (WT) human NEIL1, APE1 and their truncation mutants and the truncated polypeptides of NEIL2, OGG1, and WT SAF-A were all purified from *Escherichia coli* as described previously [33]. The expression plasmids for S59D and S59A SAF-A mutant were generated by site-directed mutagenesis (Agilent Technologies). Ku (Ku70/80 heterodimer) and DNA-PKcs were purified from HeLa cells as described previously [51].

### *In vitro* affinity co-elution assay

For the His-affinity pull down assay, His-tagged WT SAF-A or the S59D mutant was bound to Ni-NTA beads (20 μl), mixed with full-length NEIL1 or Ku in 0.5 ml TBS buffer and incubated with constant rocking for 4 h at 4°C. After washing the beads five times with 50 volume TBS buffer, the bound proteins were eluted with SDS/PAGE loading buffer and fractionated by SDS/PAGE for immunoblotting.

### *In situ* PLA

HEK293 cells were cultured in 16-well chamber slides and co-transfected with siRNA of WT SAF-A or FLAG-tagged WT SAF-A and its S59A, or S59D mutants. At 48 h following co-transfection the cells were exposed to 3Gy x-rays, were fixed with 4% paraformaldehyde, permeabilized with 0.2% Tween 20, and then incubated with the primary Ab for NEIL1 (rabbit) or FLAG-SAF-A; mouse monoclonal). The PLA assay was performed using the Duolink PLA kit (Olink Bioscience, Uppsala, Sweden) according to the manufacturer's instructions. The PLA signals were visualized in a fluorescence microscope (Olympus) at 200× magnification.

### Clonogenic survival assay

Log phase HEK293 cells were transfected with NEIL1 siRNA (80 nM, targeting the 3′ UTR region of the *NEIL1* gene; sense sequence, 5′-CCGUGAUGAUGUUUGUUUAUU-3′; antisense sequence, 5′-UAAACAAACAUCAUCACGGUU-3′; Sigma, St. Louis, MO), SAF-A siRNA (Dharmacon, catalogue number J-013501-05), APE1 siRNA (80nM, cat#SASI_Hs01_00027147, Sigma), individually or together for 48 h. Scrambled siRNA was used as a control. Downregulation of target genes was confirmed by immunoblotting of the cell extracts 48 h after transfection. Cells were irradiated with 2 Gy, 4 Gy, and 6 Gy x-rays and transferred to 6-well plates (300 cells/well) in triplicate. After allowing the cells to grow in fresh medium for 7-10 days, the colonies were stained with 0.5% crystal violet and counted.

### Affinity measurement by fluorescence spectroscopy

Interaction of SAF-A or Ku with NEIL1 C-terminal peptide (residues 312-349 lacking aromatic residues) was analysed from the change in intrinsic tryptophan fluorescence (excitation wavelength 295; emission wavelength 300-450 nm) of SAF-A or Ku, using a LS50 spectrofluorometer (PerkinElmer Life Sciences), after incubation in 10 mM PBS pH 7.5 and 5% glycerol at 25°C for 5 min. The binding constant *K_D_* was calculated by plotting Δ*F* (change in fluorescence at 345 nm) *versus* ligand concentration according to the equation Δ*F = ΔF*max × [ligand]/*K_D_ +* [ligand] [20, 52].

### Analysis of NEIL1, NEIL2, OGG1, APE1, and PNKP activity

The DNA glycosylase activities of NEIL1, NEL2, or their truncated mutants were assessed using a 5′-^32^P-labeled 5-OHU-containing oligonucleotide substrate as previously described [20, 52]. The OGG1 activity was analysed in a similar manner using an 8-OxoG containing oligo [53]. The 3′ phosphatase activity of PNKP, and the AP lyase activities of APE1 and 3′dRP were performed as previously described [35, 54].

## SUPPLEMENTARY MATERIALS


