# Supplementary material for: Scaffold attachment factor A (SAF-A) and Ku temporally regulate repair of radiation-induced clustered genome lesions
Source: Oncotarget. 2016 Jun 9;7(34):54430–44. doi: 10.18632/oncotarget.9914 (PMC5342353; doi:10.18632/oncotarget.9914)
Supplement: Supplementary file 1 [file oncotarget-07-54430-s001.pdf]

Scaffold attachment factor A (SAF-A) and ku temporally regulate repair of radiation-induced clustered genome lesions

Supplemental Material

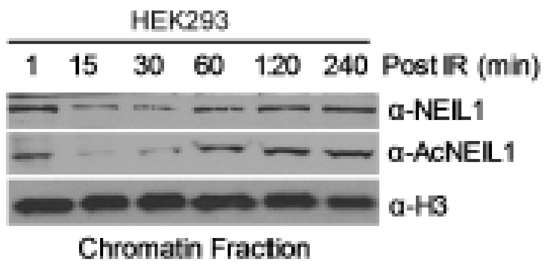

Figure S1. IR-induced release of NEIL1 from chromatin fraction correlates with its deacetylation.

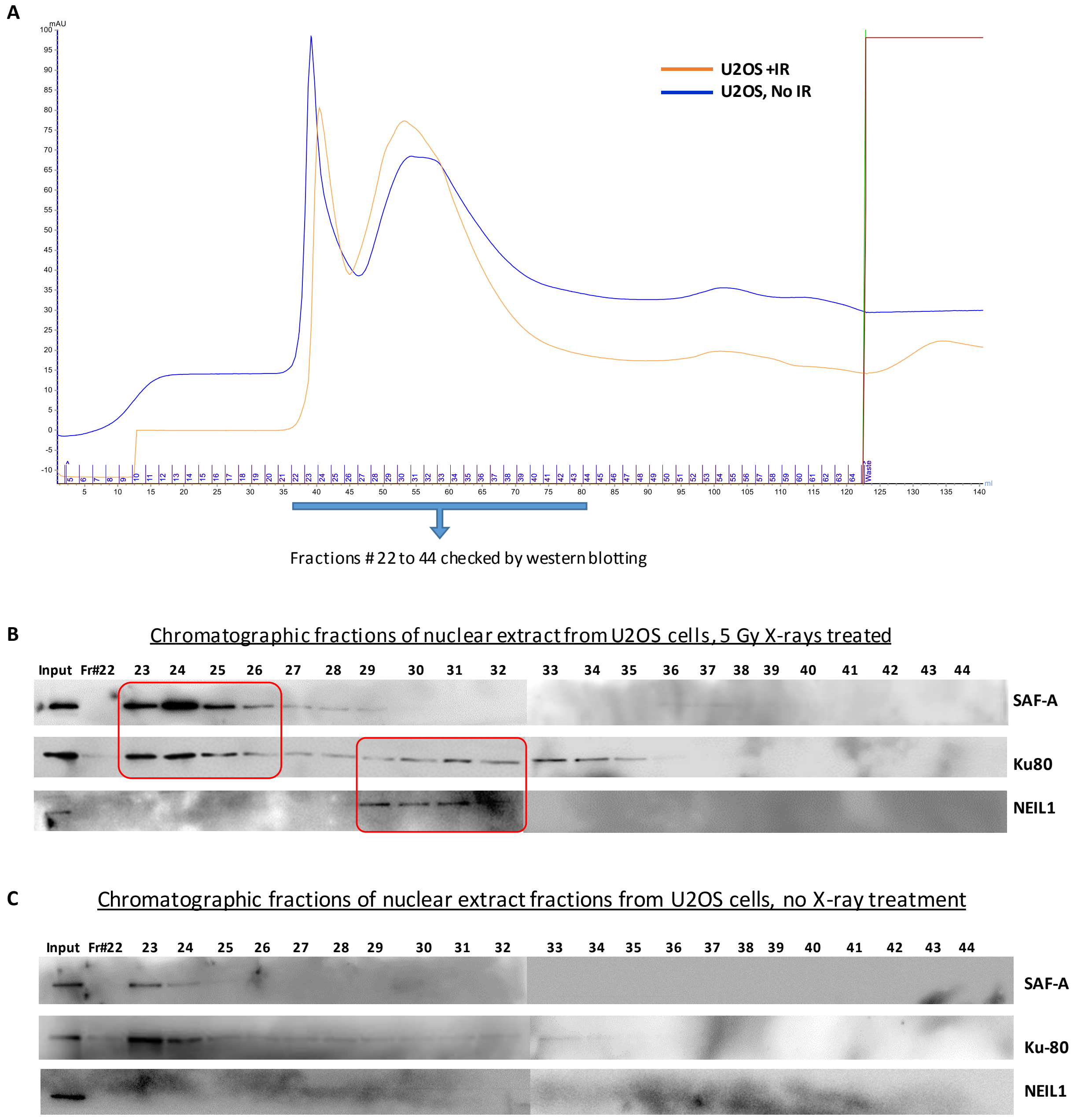

Figure S2. (A) Chromatogram for purification of U2OS cell nuclear extract, 5 Gy X-ray treated and without any treatment. 2 mg dialyzed and 0.2 micron filtered nuclear extracts were passed through HiPrep 16/60 Sephacryl S-300 HR with ÄKTA pure chromatography system (GE Healthcare Life Sciences). Fraction number 22 through 44 analyzed through western blotting, (B) western blot analysis of chromatographic fractions of nuclear extract from U2OS cells, 5 Gy X-rays treated, (C) western blot analysis of chromatographic fractions of nuclear extract from U2OS cells without any X-ray treatment; co-elution of SAF-A and NEIL1 with Ku80 have been highlighted.

A Alkaline Comet Assay

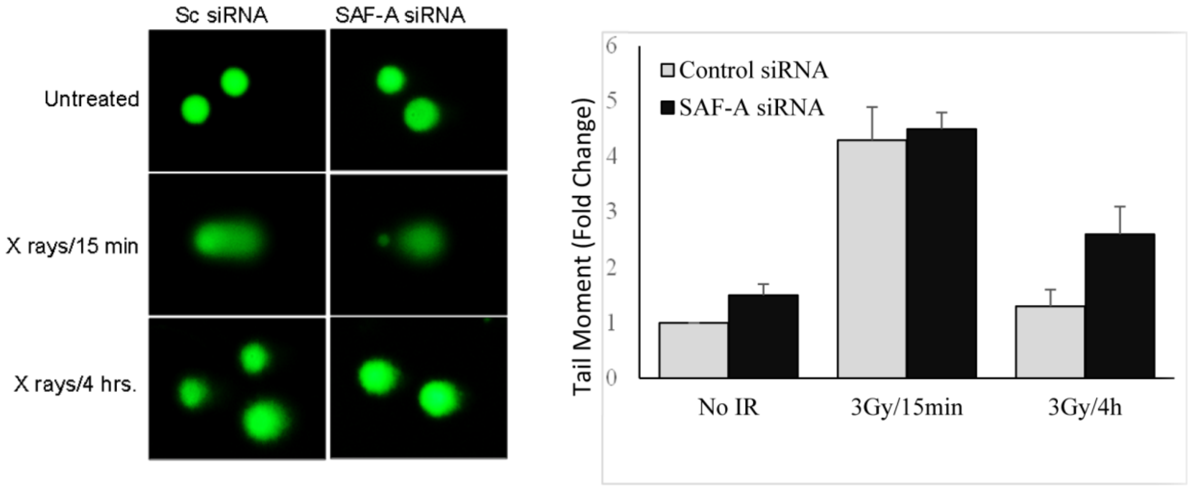

B Neutral Comet Assay

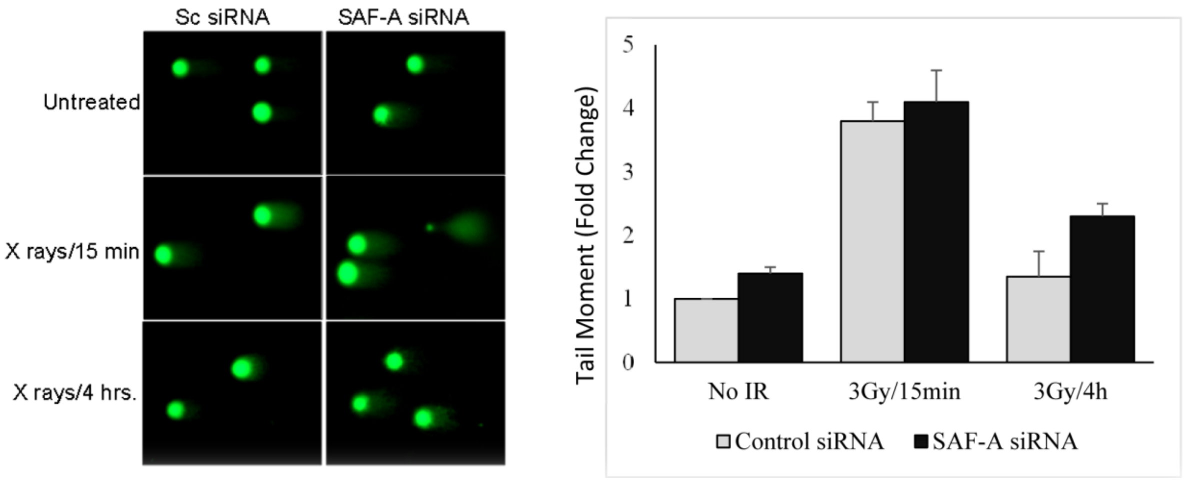

Figure S3. Comet Assay analysis of U2OS cells after SAF-A depletion. 48h after siRNA transfection, the cells were irradiated at 3Gy and subjected to alkaline (**A**) or neutral (**B**) comet assay at 15min and 4h intervals. Similar levels of unrepaired strand breaks at 4h in SAF-A depleted cells suggests that SAF-A depletion mostly leads to DSBs accumulation.
